# Interval colon cancer in a Lynch syndrome patient under annual colonoscopic surveillance: a case for advanced imaging techniques?

**DOI:** 10.1186/1471-230X-12-50

**Published:** 2012-05-24

**Authors:** Amy S Oxentenko, Thomas C Smyrk

**Affiliations:** 1 Department of Internal Medicine, Division of Gastroenterology and Hepatology, Mayo Clinic, 200 First Street SW, Rochester, MN, 55905, USA; 2 Department of Laboratory Medicine and Pathology, Division of Anatomic Pathology;Mayo Clinic, 200 First Street SW, Rochester, MN, 55905, USA

**Keywords:** Lynch syndrome, Colorectal carcinoma, Surveillance

## Abstract

**Background:**

Lynch syndrome confers increased risk for various malignancies, including colorectal cancer. Colonoscopic surveillance programs have led to reduced incidence of colorectal cancer and reduced mortality from colorectal cancer. Colonoscopy every 1–2 years beginning at age 20–25, or 10 years earlier than the first diagnosis of colorectal cancer in a family, with annual colonoscopy after age 40, is the recommended management for mutation carriers. Screening programs have reduced colon cancer mortality, but interval cancers may occur.

**Case presentation:**

We describe a 48-year-old woman with Lynch syndrome who was found to have an adenoma with invasive colorectal cancer within one year after a normal colonoscopy.

**Conclusion:**

Our patient illustrates two current concepts about Lynch syndrome: 1) adenomas are the cancer precursor and 2) such adenomas may be “aggressive,” in the sense that the adenoma progresses more readily and more rapidly to carcinoma in this setting compared to usual colorectal adenomas. Our patient’s resected tumor invaded only into submucosa and all lymph nodes were negative; in that sense, she represents a success for annual colonoscopic surveillance. Still, this case does raise the question of whether advanced imaging techniques are advisable for surveillance colonoscopy in these high-risk patients.

## Background

Lynch syndrome is defined as the presence of a germline mutation in a DNA mismatch repair gene [1]. Mutation carriers have increased risk for various malignancies, including carcinomas of the colorectum (CRC), endometrium, ovary, small bowel, stomach, biliary tract, bladder, ureter and renal pelvis [2]. When a mutation is documented in an affected individual, genetic counseling and mutation analysis should be offered to related family members. Mutation carriers face a range of difficult decisions regarding prophylaxis and surveillance. Prophylactic hysterectomy, for example, offers low morbidity and guaranteed protection against endometrial cancer, but may conflict with the desire to bear children [3]. Prophylactic colectomy is also an option, but regular colonoscopic examination is safe and effective and is the favored approach for most patients [4].

Colonoscopic surveillance should begin at age 20–25. There has been debate about the optimal timing for examinations, with the recommended interval narrowing recently from three years to two years and finally to the current preference for colonoscopy every one to two years until age 40, with yearly colonoscopy thereafter [5]. The rationale for the one-year timeframe is that interval cancers have been described in patients being examined every two or three years, probably in part because the precursor lesion for CRC in Lynch syndrome – the adenoma – progresses through the adenoma-carcinoma sequence more rapidly than a sporadic adenoma.

We describe a young woman with known Lynch syndrome who presented for her surveillance colonoscopic examination and was discovered to have a right-sided CRC arising in a tubular adenoma, one year after a normal examination.

## Case presentation

**Figure 1:**
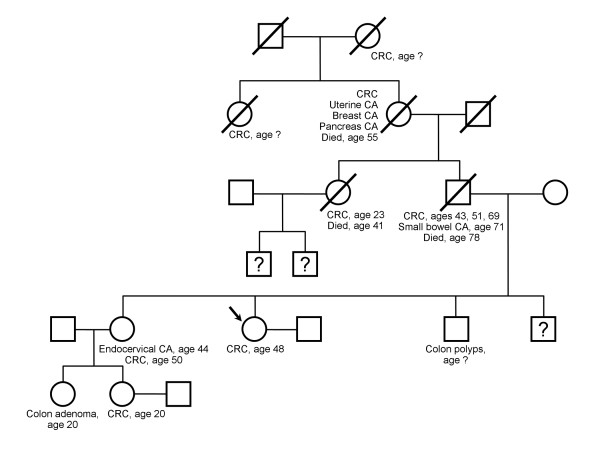
**Family pedigree in the described patient with Lynch syndrome.** Arrow indicates the presented patient. CRC = colorectal cancer; CA = cancer; ? = unknown history.

The patient is a 48-year-old female with known Lynch syndrome. Her father, the proband, had primary CRC at age 43, 51 and 69, then a jejunal adenocarcinoma at age 71. He was found to have a truncating mutation on MLH1 (IVS7-2A > G) and a separate missense mutation of uncertain significance (V716M). The family tree is replete with other malignancies characteristic of Lynch syndrome (Figure 1).

Based on family history alone, the patient began colonoscopic evaluations every five years beginning at age 18. She had genetic testing at age 42 and was found to carry both the deleterious truncating mutation and the missense mutation. She underwent prophylactic hysterectomy and bilateral salpingo-oophorectomy, and began a program of annual colonoscopy in 2003.

**Figure 2:**
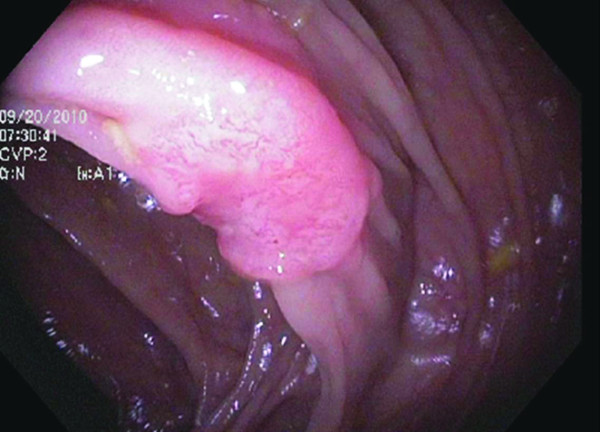
Endoscopic photo demonstrating a 1.2 cm lesion on top of a fold in the ascending colon.

**Figure 3:**
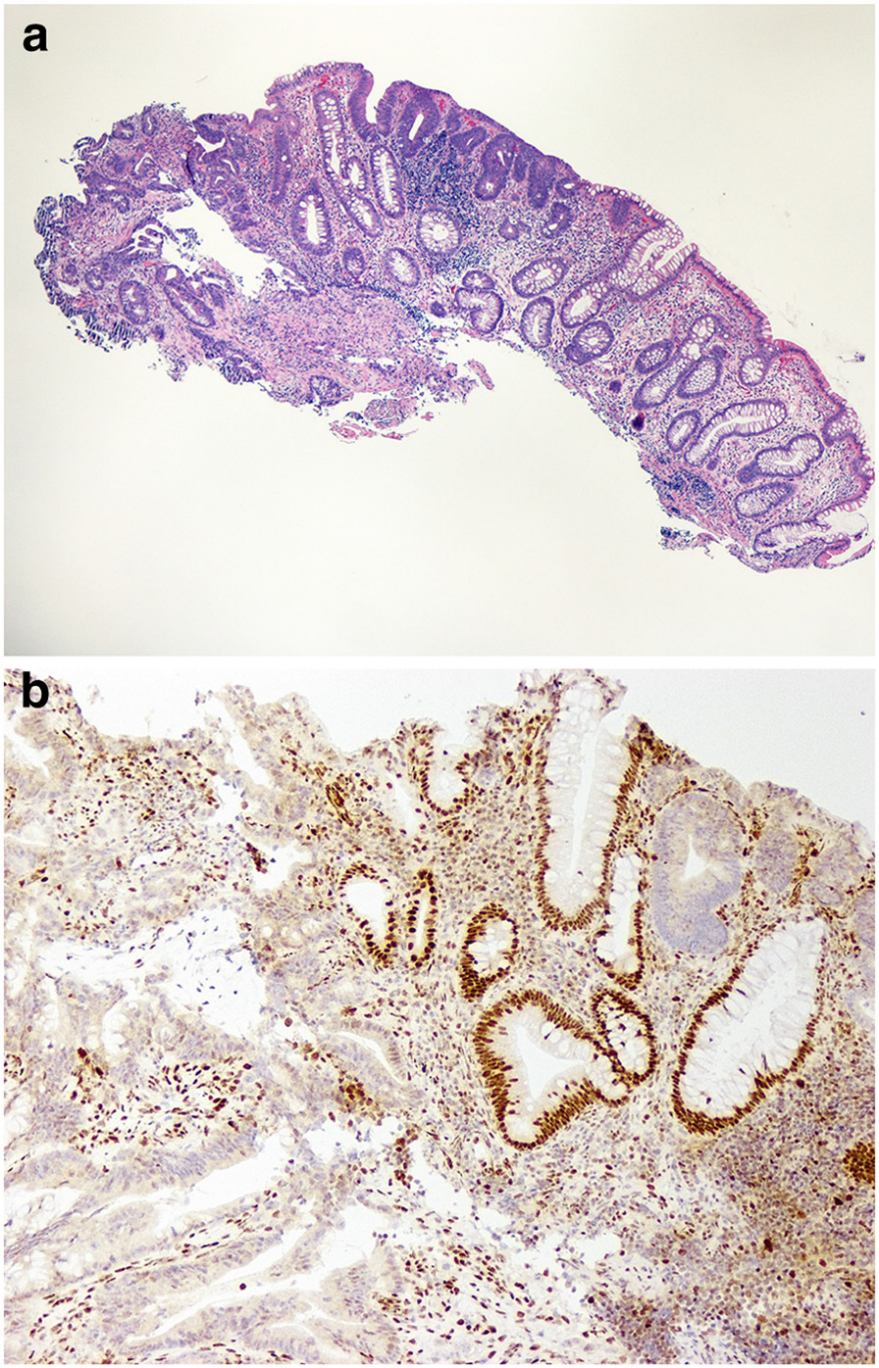
**a) Biopsy of mucosal lesion, showing adenoma (right) and carcinoma (left).** (Original magnification 40x) **b**) Immunohistochemistry for MLH1, performed on the endoscopic biopsy. The adenoma and the cancer both show loss of expression. Non-neoplastic crypts in the center of the field show the expected nuclear staining. (Original magnification 100x).

In September 2009, the patient established her cancer surveillance at our institution. Her past medical history was significant only for migraine headaches, hypothyroidism and endometriosis. She was married, with no children, had no tobacco history and infrequent alcohol use. Her physical examination was unremarkable. Laboratory studies at that time were all within normal limits, including hemoglobin of 12.3 g/dL. Full colonoscopy with intensive inspection was performed and was negative, with good bowel preparation noted. The remainder of her tests, including mammography, upper endoscopy, CT enteroclysis and urine cytology were all negative or normal. The patient had no symptoms referable to her lower GI tract over the ensuing year. At her September 2010 exam, laboratory studies revealed hemoglobin of 11.8 g/dL with a mean corpuscular volume of 81.5 fL (normal 81.6-98.3 fL). At colonoscopy, the endoscopist noted “a 1.2 cm, malignant-appearing flat lesion on top of a fold” in the ascending colon (Figure 2). Biopsy showed a tubular adenoma adjacent to a moderately differentiated adenocarcinoma. Immunohistochemistry for MLH1 demonstrated loss of expression in both the adenoma and the carcinoma (Figure 3). Her carcinoembryonic antigen (CEA) level was 0.5 ng/mL (normal ≤ 3.0 ng/mL). Eight days later, she had a hand-assisted subtotal colectomy with ileorectostomy. The resection specimen contained a 1.8 cm, moderately differentiated adenocarcinoma arising 7.5 cm distal to the ileocecal valve. An adenoma extended from one edge of the cancer for a distance of 9 mm. The tumor invaded into the submucosa but not into the muscularis propria, and multiple (49) lymph nodes were negative, making this a T1N0 lesion. There were no other mucosal lesions. The post-operative course was uneventful. She was discharged 7 days after her operation. Follow-up examination and surveillance testing one year later were unremarkable.

## Conclusions

Lynch syndrome is not rare. The magnitude of its contribution to the total CRC burden has been debated, with some estimates as high as 6% [6] and others as low as 0.86% [7]. Current estimates are that approximately 3% of all CRCs arise in the setting of Lynch syndrome [8,9]. Surveillance programs conducted in Europe during the late 20^th^ century put the lifetime risk for CRC at 70-80% [10,11]. That number has been a matter of some controversy.

A large study which applied corrections for ascertainment bias calculated a lifetime CRC risk of only 27% for men and 22% for women [12]. But other large studies, also controlled for ascertainment bias, have arrived at lifetime CRC risks of 45-66% for men and 38-43% for women [13]. In some reports, the risk for CRC is modulated by the gene involved (higher for MLH1) [13], sex (higher for men), and possibly environmental exposures (risk reduced by dietary fruit and fiber, increased by cigarette smoking) [14]. It is also likely that the specific mutation influences cancer risk; indeed, a criticism of one early risk study [11] was that the large majority of the study patients carried the same founder mutation on MLH1, and were thus not representative of the full spectrum of Lynch syndrome patients. As more data is accumulated, it should become possible to individualize patient risk based on all of the above factors.

Adenoma is the precursor lesion for CRC in Lynch syndrome. The original term “hereditary non-polyposis colorectal carcinoma (HNPCC)” was coined to separate the condition from polyp-forming syndromes, particularly familial adenomatous polyposis. While patients with Lynch syndrome don’t have large numbers of adenomas, they clearly form them more readily than the general population [15,16]. Mecklin et al. found the cumulative incident risk for adenoma to be 68% in men and 48% in women by age 60 [16]. The authors used their data to calculate that 40% of men and 30% of women have a colorectal neoplasm of some kind by age 40. In contrast, only 1-2% of the general population has an adenoma at that age [17].

Regular endoscopic surveillance aimed at discovering and removing adenomas reduces mortality from CRC in Lynch syndrome. A controlled trial among 252 Finnish patients deemed to be at 50% risk of CRC by virtue of family history demonstrated a 62% reduction in CRC among those who had regular surveillance compared to those who refused surveillance [18]. Tumor stage was significantly more favorable in the study group, and none of 133 patients under surveillance died of CRC, compared to 9 of 119 (8%) of controls. More recently, de Jong et al. described a long-term surveillance program in the Netherlands and showed a 70% reduction in the standard mortality ratio for CRC during the surveillance era [19].

There is theoretical evidence that the adenomas of Lynch syndrome can progress to carcinoma more rapidly than sporadic adenomas. Immunohistochemistry generally shows loss of staining for mismatch repair proteins in adenomas from Lynch syndrome patients [20,21], meaning that they have already lost the ability to repair DNA mismatches. The very rapid rate at which mutations accumulate in this setting provides a molecular mechanism for the “aggressive adenoma” concept proposed many years ago [22-24].

A recent study involving 54 patients with known mutations in MLH1 or MSH2 gives strong observational support to the aggressive adenoma idea [25]. Surveillance by colonoscopy every 1–2 years over a mean follow-up period of 9.3 years led to the detection of 112 adenomas and 31 CRC. The authors calculated a polyp dwell time (the time interval from normal mucosa to CRC) of 35.2 +/− 22.3 months. Admittedly, the standard deviations are large, but the numbers are strikingly shorter that the 10 years estimated for the adenoma-carcinoma sequence in sporadic CRC [26].

What is the proper surveillance interval? Numerous large-scale surveillance programs support the notion of rapid carcinogenesis. Jarvinen et al. achieved their reduction in CRC using colonoscopy every three years, but interval cancers did occur under that strategy and others like it [16,18,27]. Vasen et al. showed that for carriers of MLH1 or MSH2 mutations, a surveillance interval of 1–2 years reduced CRC risk compared to 3-year intervals [28]. A multicenter cohort study from Germany found good compliance to a regimen of annual surveillance colonoscopy among 1126 individuals; that prospective study, using standard video colonoscopy, detected 43 incident CRC, 19 of which had been preceded within 12 months by a normal colonoscopy [29].

Does our patient’s tumor represent a rapidly developing malignancy or a missed polyp (or both)? Missed polyps are an unfortunate reality; one prospective multicenter study found a miss rate for adenomas of 20% [30]. Looking specifically at Lynch syndrome, Stoffel et al. found a stunningly high adenoma miss rate of 55% when standard colonoscopy was followed by a tandem second exam with chromoendoscopy and/or intensive inspection [31]. The fact that the neoplasia in Lynch syndrome is often right-sided might contribute to this; the polyp dwell time in Edelstein’s study was shorter for right-sided lesions (28.7 months) than for those in the left colon (43.6 months) [25]. The differences were not statistically significant, but they do hint that both rapid tumor growth and missed adenomas contribute to the problem of interval cancers in Lynch syndrome. The issue of rapid tumor growth can be addressed by emphasizing the importance to patients and providers of adhering to a strict annual surveillance program. Accountability to various quality metrics, such as withdrawal time and adenoma detection rates, could help decrease missed adenomas by endoscopists. Finally, risk variations within families could conceivable alter the aggressiveness with which one pursues surveillance; our patient’s sister was diagnosed with a T3N0M0 colon cancer one year after a normal annual surveillance colonoscopy, having received annual examinations over a number of years at our institution as well. While it is possible that there could have been missed lesions in both cases, we think it more likely that something in the genetic/environmental background of this family makes them avid carcinoma formers.

Our patient was not in a research study. Her colonoscopies were conducted by experienced gastroenterologists who were aware of her mutation status. She was given a split-dosed bowel preparation for both her 2009 and 2010 colonoscopies, and intensive inspection was performed; narrow-band imaging was available, but no additional visualization techniques such as chromoendoscopy were used. Standard video colonoscopy has been the technique of choice in large-scale surveillance programs [29] and current surveillance recommendations do not include the use of enhanced visualization methods [4]. Although Stoffel et al. reported that the high adenoma miss rate could be corrected by EITHER chromoendoscopy or intensive inspection at the second-look procedure [31], others have found that the use of chromoendoscopy may indeed enhance adenoma detection compared to colonoscopies performed with standard white light or narrow band imaging technique [32]. Additional studies are required to see whether adding chromoendoscopy to standard intensive inspection in patients with Lynch syndrome confers reproducible benefit, perhaps supporting its inclusion in future cancer surveillance guidelines for this patient population.

This report highlights several features of Lynch syndrome. First, individuals carrying deleterious mutations in a DNA mismatch repair gene (MLH1, MSH2, MSH6, PMS2) deserve annual colonoscopic examinations with a careful search for, and removal of, all mucosal lesions. Second, adenoma is the precursor lesion for CRC in Lynch syndrome. Such adenomas are “aggressive,” in the sense that the time from normal mucosa to CRC is much shorter than in the general population; close follow-up of patients with Lynch syndrome is essential for this reason. Next, screening colonoscopy works, and it works in the setting of routine clinical practice. There has been some concern that lessons learned from mass screening programs might not transfer to daily practice [33]. However, our patient and multiple other members of her family are cared for by a single gastroenterologist who does not have a specific research interest in Lynch syndrome. It may take encouragement to get patients who feel well to adhere to the somewhat onerous requirements of a strict surveillance regimen, but that is part of a successful physician-patient relationship, and its rewards are illustrated by this report. Finally, the incorporation of advanced imaging techniques into surveillance guidelines for patients with Lynch syndrome undergoing annual colonoscopic examinations will likely evolve if additional studies can demonstrate consistent benefit, which may be especially important in patients with high-risk features.

## Consent

Written informed consent was obtained from the patient for publication of this Case report and any accompanying images. A copy of the written consent is available for review by the Series editor of this journal.

## Competing interests

The authors declare that they have no competing interests.

## Authors’ contributions

ASO is the patient’s physician. She helped draft the manuscript and prepare figures. TCS helped draft the manuscript and prepare figures. All authors read and approved the final manuscript.

## Pre-publication history

The pre-publication history for this paper can be accessed here:

http://www.biomedcentral.com/1471-230X/12/50/prepub
